# Both Ca^2+ ^and Zn^2+ ^are essential for S100A12 protein oligomerization and function

**DOI:** 10.1186/1471-2091-10-11

**Published:** 2009-04-23

**Authors:** Olga V Moroz, Will Burkitt, Helmut Wittkowski, Wei He, Anatoli Ianoul, Vera Novitskaya, Jingjing Xie, Oxana Polyakova, Igor K Lednev, Alexander Shekhtman, Peter J Derrick, Per Bjoerk, Dirk Foell, Igor B Bronstein

**Affiliations:** 1 Department of Chemistry, University of York, York, UK; 2 Department of Chemistry, University of Warwick, Coventry, UK; 3 Department of Pediatrics and Interdisciplinary Center for Clinical Research, Institute of Immunology, University of Muenster, Muenster, Germany; 4 Department of Chemistry, University at Albany, SUNY, Albany, NY, USA; 5 Department of Chemistry, Carleton University, Ottawa, Canada; 6 University of Maryland Biotechnology Institute, Baltimore, MD, USA; 7 MRC National Institute for Medical Research, Mill Hill, London, UK; 8 Active Biotech Research AB, Lund, Sweden; 9 Wolfson Centre for Age-Related Diseases, School of Biomedical and Health Sciences, King's College London, London, UK

## Abstract

**Background:**

Human S100A12 is a member of the S100 family of EF-hand calcium-modulated proteins that are associated with many diseases including cancer, chronic inflammation and neurological disorders. S100A12 is an important factor in host/parasite defenses and in the inflammatory response. Like several other S100 proteins, it binds zinc and copper in addition to calcium. Mechanisms of zinc regulation have been proposed for a number of S100 proteins e.g. S100B, S100A2, S100A7, S100A8/9. The interaction of S100 proteins with their targets is strongly dependent on cellular microenvironment.

**Results:**

The aim of the study was to explore the factors that influence S100A12 oligomerization and target interaction. A comprehensive series of biochemical and biophysical experiments indicated that changes in the concentration of calcium and zinc led to changes in the oligomeric state of S100A12. Surface plasmon resonance confirmed that the presence of both calcium and zinc is essential for the interaction of S100A12 with one of its extracellular targets, RAGE – the Receptor for Advanced Glycation End products. By using a single-molecule approach we have shown that the presence of zinc in tissue culture medium favors both the oligomerization of exogenous S100A12 protein and its interaction with targets on the cell surface.

**Conclusion:**

We have shown that oligomerization and target recognition by S100A12 is regulated by both zinc and calcium. Our present work highlighted the potential role of calcium-binding S100 proteins in zinc metabolism and, in particular, the role of S100A12 in the cross talk between zinc and calcium in cell signaling.

## Background

S100A12 and other proteins of this family have been implicated in the regulation of a wide range of physiological and pathophysiological processes [1-3].

Human S100A12 was discovered in blood cells [4]. It was estimated that it constituted about 5% of total cytosolic protein in resting neutrophils. Soon after this discovery the first data on S100A12 functional activity were reported. A calgranulin-related protein (CGRP) was purified from the extracts of the human parasite *Onchocerca volvulus *[5]. A search for S100A12 binding sites on another helminth, *Brugia malayi*, resulted in the identification of paramyosin, a muscle protein localized just below the parasite's cuticle [6]. A further search of S100A12 targets led to the discovery of RAGE – the Receptor of Advanced Glycation End products [7]. RAGE is a pattern recognition receptor and a multiligand member of the immunoglobulin superfamily of cell surface adhesion molecules [8,9]. RAGE is linked to cellular dysfunction in several inflammatory disorders, in tumors and in diabetes [10-12]. It was proposed that the interaction of RAGE with S100A12 mediates proinflammatory effects on lymphocytes and mononuclear phagocytes [7]. Existence of another receptor, different from RAGE, was suggested in the recent report on the effect of S100A12 and its "hinge" peptide on mast cell and monocyte recruitement. Interactions with an as yet unidentified G-protein coupled receptor were proposed [13]. These and other data indicate that extracellular S100A12 interactions with its diverse targets may contribute to the pathogenesis of many diseases and inflammatory responses [14-16] including some neurodegenerative disorders [17]. High-resolution structural data of RAGE/S100 complex are still unavailable. However, a significant impact on the mechanism of these interactions has been made by X-ray crystallography of S100A12 protein [18,19] and NMR studies of RAGE/S100A12 complex [20].

A few intracellular targets of S100A12 namely cytosolic NADP+-dependent isocitrate dehydrogenase (IDH), fructose-1,6-bisphosphate aldolase A (aldolase), glyceraldehyde-3-phosphate dehydrogenese (GAPDH), annexin V and S100A9 have also been detected [21]. The ability of S100A12 for translocation to the cellular membrane and its secretion [4,22] raises a question whether some of its targets are able to form a complex with a transport function. These putative "S100 transporters" are still unknown. However S100A13 multiprotein secreted complex has recently been identified [23].

Several S100 proteins bind zinc. Two major types of zinc-binding sites (with and without cysteine residues) have been identified. The cysteine free Zn-binding site was fully characterized from the 3D structure of S100A7 [24] and S100B [25]. Different zinc-binding motifs containing cysteine were suggested for S100A2 based on the NMR data and homology modeling [26]. S100 proteins were characterized by a wide range of zinc binding constant (S100B [Kd 90 nM], S100A2 [Kd 25 nM], S100A3 [Kd 1.5 μM], S100A5 [Kd 2 μM], S100A6 [Kd 0.1 μM] and S100A7 [Kd 100 μM]), and in each case a conformational change was observed upon binding of Zn^2+ ^ions [24-30]. Zinc binding constant for pig S100A12 was estimated from fluorescence titration curves (Kd < 10 nM), binding of zinc caused a large increase (~1500-fold) in its affinity for Ca^2+ ^[31]. An opposite effect has been shown for S100A2 protein. Recent findings suggest that Zn^2+ ^might deactivate S100A2 by inhibiting response to intracellular Ca^2+ ^signals [32]. The role of Zn in S100 Ca binding proteins is so far not clear. However, evidence of the influence of zinc on the levels of the intracellular calcium started to emerge. In particular, changes in the extracellular zinc concentrations triggered a release of calcium from the intracellular pools in colonocytic cell line HT29, so the presence of an uncharacterized zinc-sensing receptor was proposed [33]. In another study, zinc inhibited calcium influx by blocking store-operated calcium channel (SOCC) in human salivary gland cells suggesting redox-dependent regulation [34]. Despite its clinical importance little is known about cellular signaling mechanisms that sense changes in extracellular zinc concentration. A missing link between extracellular zinc and regulation of cellular physiological processes might be an important cellular transduction pathway in the cross talk between the cell and its microenvironment. Although the total zinc concentration in serum is about 15 μM [35,36], almost all of that is bound to the proteins, with a very wide range of zinc affinity from picomolar to micromolar levels, which means that zinc is exchangeable and this exchange depends on the local concentration of zinc binding proteins with different Zn^2+ ^affinity.

Our present work draws attention to a potentially significant role of S100 proteins in Zn metabolism. Zn binding to S100A12 protein provides a greater diversity of protein conformational changes and therefore may dramatically modulate its function as a result of changes in the cellular microenvironment.

## Results

### S100A12 binds to the cellular surface in a zinc dependent manner

The viability and growth of tissue cell culture cell lines depend on the presence of metal ions in a cell culture medium. Fluctuations of extracellular zinc appear to be a significant aspect of cellular signal transduction. Several well established tissue cell culture mediums contain remarkably different concentrations of divalent cations such as calcium, zinc and copper (see Methods). Though the experiments with cells exposed to Zn or Cu are sometimes difficult to interpret numerous data indicate a target upstream of the intracellular signaling pathways.

**Figure 1:**
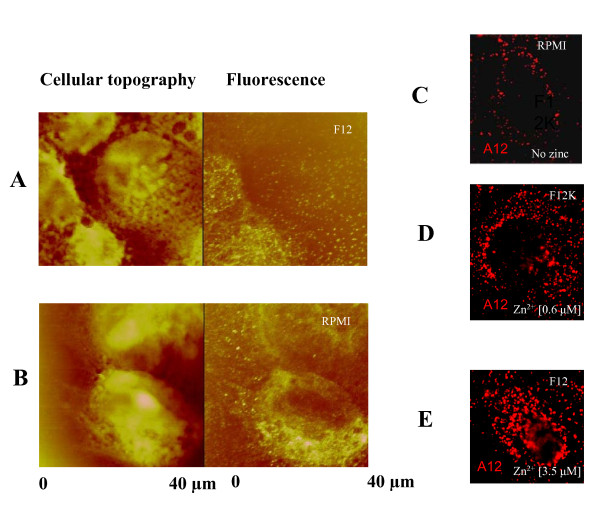
**NSOM topography and fluorescent images of MKN74 cells treated with S100A12**. in F12 (**A**) and RPMI 1640 (**B**) cell media. Major fraction of the clusters detected has an intensity of ~2 MHz (million counts per second). Fractions with intensities ~4, 8, and 10 MHz were also detected suggesting the presence of S100 aggregates greater than hexamers. Total number of clusters detected was ~500/image. (**C**-**E**) Images of gastric carcinoma MKN74 cells captured by epifluorescence microscopy. Cells were incubated in the presence of S100A12 protein in RPMI (**C**), F12K (**D**) and F12 (**E**) cellular mediums without serum. Cells were stained with affinity-purified rabbit anti-S100A12.

We analyzed the binding of S100A12 to the cellular surface of human gastric carcinoma MKN74 cells in RPMI-1640 and F12 medium. This cell line expresses an elevated level of endogenous RAGE [37]. To reduce the rate of endocytosis the binding reaction was performed at 10°C or at room temperature. We demonstrated that S100A12 could be detected on the cellular surface by using near field scanning optical microscopy (NSOM). Remarkably the S100A12 binding in F12 medium containing zinc showed a different binding pattern compared to that of zinc depleted RPMI-1640 medium (Fig. 1A, B). Morphologically many bright speckles (0.6 clusters/square micrometer) of fluorescence were detected on the cell surface in the F12 zinc containing medium. In the RPMI-1640 medium the number of fluorescent dots were markedly decreased to less than 0.2 clusters/square micrometer. We suggest that incubation of MKN74 cells in the Zn containing medium in the presence of exogeneous recombinant S100A12 led to the more efficient binding of S100A12 to cell surface receptors or other molecules. (Fig. 1A, B). Although these results do not allow us to distinguish between the various dimeric, tetrameric and hexameric structures suggested by X-ray analysis and other analytical techniques, it is obvious that clustering and assembly of S100A12 with extracellular receptors in the presence of zinc are significantly enhanced. It is very likely that oligomerisation occurs in the solution and oligomers are then trapped on the cellular surface as large clusters in the range of 100–400 nm. We can suggest that such observed clustering is a combined result of S100A12 and receptor aggregation. It was intriguing to speculate that S100A12 cellular binding activity could be a property associated with protein conformational changes mediated by the action of both Ca and Zn ions.

These results were also confirmed by confocal fluorescent microscopy (Fig. 1C, D, E). Both sets of data provide evidence that the presence of Zn in cell culture medium is important for S100A12 oligomerization and its binding to cell surface molecules. Cellular assays at this stage of research were postponed due to a very complicated interpretation of S100A12 binding targets. It was shown that different extracellular molecules such as heparin sulphate proteoglycans were able to bind S100A12 and other S100 proteins [38]. RAGE receptor – the primary S100A12 target – is not uniformly glycosylated *in vivo *and only a small proportion of RAGE molecules are able to form a productive complex with its ligands [39,40], (Srikrishna & Freeze, unpublished results). Therefore our further experiments were focused on *in vitro *studies to demonstrate that zinc/calcium-dependent oligomerization of S100A12 may be an additional and very important factor contributing to its target interactions; this was probed on the example of the best known interaction partner, RAGE.

### Surface plasmon resonance

**Figure 2:**
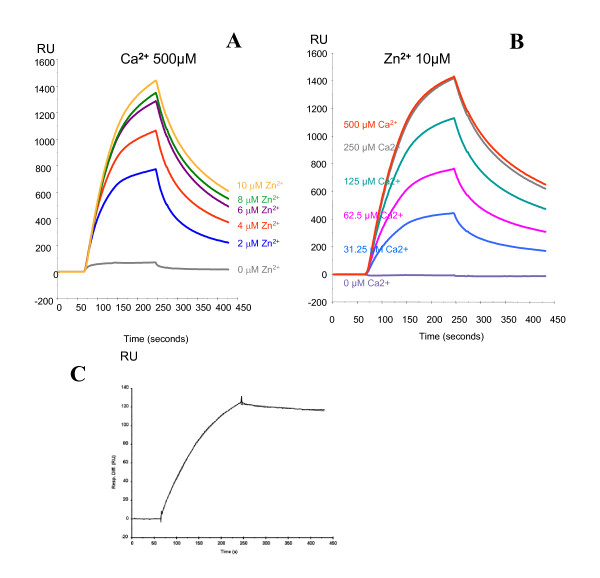
**Surface plasmon resonance analysis of S100A12-RAGE interaction**. (**A **and **B**). Sensorgrams on Ca^2+^- and Zn^2+^-dependent binding of S100A12 to immobilized RAGE. Sensorgrams show association and dissociation with varying conditions in which either Ca^2+ ^was kept stable with varying Zn^2+ ^concentrations or vice versa. One representative out of three independent experiments is shown as response units (RU; y-axis). Time is shown in seconds. (**C**). Kinetic and affinity analysis of RAGE binding to immobilized S100A12. Recombinant human RAGE/Fc (125 nM) was injected over a surface where recombinant S100A12 was immobilized at a density of 2,933 RU on a CM5 chip using amine coupling chemistry. Samples were injected for 3 min at a flow rate of 30 μl/min. HBS-P buffer (Biacore) containing 1 mM Ca^2+ ^and 20 μM Zn^2+ ^was used as sample and running buffer. The sensorgram obtained after injection of 125 nM RAGE (straight line) was perfectly fit to a 1:1 Langmuir model (dotted line; BIAevaluation software; Biacore) for calculation of affinity and on-/off-rates. The K_D_, kon, koff and Chi^2 ^values were calculated to: 4.6 × 10-9 M; 6.8 × 104 1/Ms; 3.2 × 10-4 1/s; and 0.47, respectively.

We have exploited surface plasmon resonance technique to analyze the binding of S100A12 to immobilized RAGE with different Ca and Zn concentrations in greater detail *in vitro*. Our results show that the interaction of these molecules depends both on Ca and Zn (Fig. 2A–C). More detailed studies on affinity and on/off-rates could not be calculated from these SPR curves as they do not fit into any reliable binding model. Therefore we used immobilized S100A12 to show affinity of the S100A12/RAGE interaction, which is in the nanomolar range [4.6 × 10^-9 ^M], and calculated kon [6.8 × 10^4 ^1/(M×s)], koff [3.2 × 10^-4 ^1/s] and Chi^2 ^[0.47] values after fitting the response curve to a 1:1 Langmuir model.

The most likely explanation for the differences in the SPR results for binding of RAGE to immobilized S100A12 vs that of S100A12 to immobilized RAGE lies in the different ways the proteins are attached to the surface. Because we used amine coupling, we are unable to say for sure how exactly each of the proteins was attached. We can suppose, however, that in the case of S100A12-to-RAGE there was more hindrance of the immobilized protein (in other words – the recognition site was partially obstructed). Moreover, each of the proteins could be attached by a number of different sites, and this possibly complicated the analysis of the results for the case of immobilized RAGE. Nevertheless, the main aim of the study, namely, showing the effect of calcium and zinc on the interaction between S100A12 and RAGE has been achieved.

### Translational Diffusion of S100A12 in the presence of Zn and Ca ions

**Figure 3:**
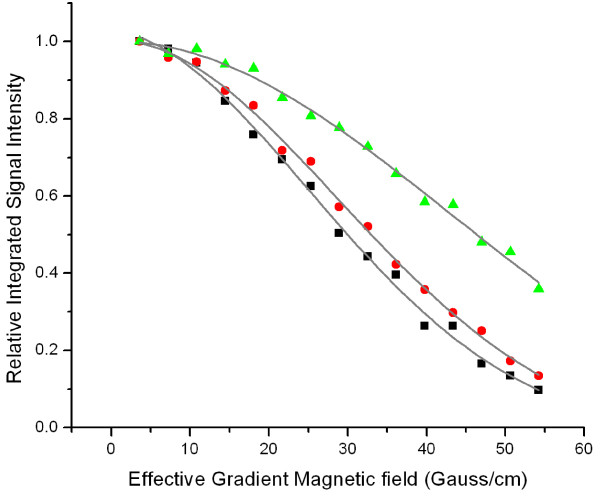
**Decay of the integrated NMR signal intensity**. of the apo-S100A12 (squares), Ca^2+^-S100A12 (circles), and Zn^2+^, Ca^2+^-S100A12 (triangles) as a function of the magnetic field gradients. Proton NMR signal was integrated over the amide region (from 6.5 ppm to 10.5 ppm). The translational diffusion coefficients for the apo-S100A12, Ca^2+^-S100A12, and Zn^2+^, Ca^2+^-S100A12 determined by curve fitting to equation 1, were 1.12 ± 0.07·10–10 m2/s, 0.95 ± 0.06·10–10 m2/s., and 0.53 ± 0.07·10–10 m2/s, respectively.

Large biomolecular oligomers scatter light disproportionally stronger than the monomers resulting in a dynamic light scattering (DLS) bias towards multimers. Solution NMR experiments are more sensitive to the smaller oligomers, thus, presenting a complimentary approach to DLS. Pulsed field gradient NMR experiments were used to measure translational diffusion coefficient of the apo-form of S100A12 and its divalent complexes with Ca^2+ ^and Zn^2+ ^and Ca^2+^, respectively. Fitting the experimental data (Fig. 3) to the theoretical dependence of the signal attenuation on the strength of the gradient field (Eq 6 and 7), we obtained the values for the translation diffusion coefficients for apo-, Ca^2+^, and Ca^2+^, Zn^2+ ^forms of S100A12 of 1.12 ± 0.07·10^-10 ^m^2^/s, 0.95 ± 0.06·10^-10^m^2^/s, and 0.53 ± 0.07·10^-10^m^2^/s, respectively. According to (Eq 5), ratio between translational diffusion coefficients is inversely proportional to the ratio of hydrodynamic radii of the oligomers. Based on the NMR data we have obtained the value for the ratio between radii of 1:1.18:2.11 for apo-S100A12, Ca^2+^-S100A12, and Zn^2+^, Ca^2+^-S100A12 respectively. This value can be directly compared to the ratio derived from the DLS data 1:1.25:3.09 (data not shown). The difference between the DLS and NMR results suggests that binding of divalent ions to S100A12 produces a complex mixture of exchanging oligomers in solution. Both the size and the relative population of the oligomers are increasing with the addition of Zn ions.

### Native PAGE

**Figure 4:**
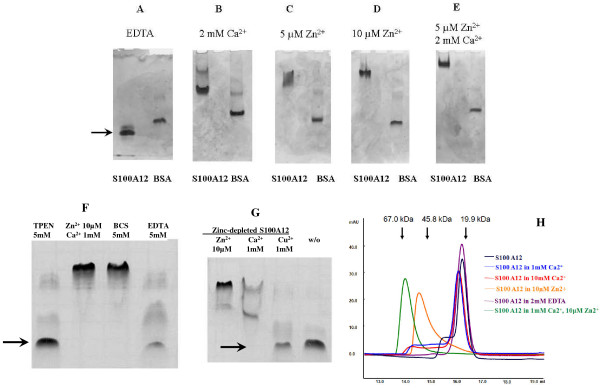
**Native PAGE and overlay of size-exclusion chromatograms of S100A12 in the presence of EDTA and different cations**. Loading concentration of S100A12 – 14 μg (**A**-**E**). Dimeric S100A12 is indicated by an arrow. Concentrations of zinc acetate and calcium chloride are indicated above the gel picture. (**F**) TPEN (zinc-chelator), BCS (copper I-chelator), or EDTA (divalent cation chelator) was added. After removal of calcium or zinc from the buffer solution the dimeric S100A12 is the strongly preferred form, while removal of copper did not produce a measurable effect. (**G**) S100A12 in a zinc- and calcium-free solution was re-substituted with zinc, copper, or calcium before loading the gel. There were no hexamers in the absence of zinc (w/o), which is also not influenced by addition of copper. When re-substituting zinc, there is predominantly a hexamer formation, which is also induced to a lesser extent by adding calcium, where a second band eventually representing tetrameric S100A12 can be observed. (**H**) SEC was performed using Akta FPLC chromatography equipment (Amersham Pharmacia Biotech) and a Superdex 200 (10 mm×300 mm) gel-filtration column (Amersham Pharmacia Biotech). Before each run the column was equilibrated with elution buffer 50 mM Tris, pH 7.5, 200 mM NaCl. The flow rate was 0.5 ml/minute and the protein elution was monitored by UV-absorption at 280 nm. S100A12 Abs 280/260 = 0.4/0.3. S100A12 elutes as a dimer in the presence of EDTA (shown in violet), shows a tendency to oligomer formation at increasing calcium concentrations (red and blue curves), elutes as a tetramer in the presence of zinc (orange), and as a hexamer with calcium and zinc (green).

Analysis of relative electrophoresis mobilities in non-denaturing gels provided a sensitive technique to determine the oligomerization state of the protein and estimate molecular masses of multiple species. At least two fast migrating bands could be resolved when apo-S100A12 was electrophoresed through the native gel (Fig. 4A). In contrast, slower migrating bands could be detected when the same amount of the protein was run in 2 mM Ca (Fig. 4B), these possibly corresponded to the tetramers and a much smaller amount of hexamers. In the presence of 5 μM Zn the bands possibly corresponded to a tetramer-hexamer transition (Fig. 4C) and with 10 μM Zn – hexamers or some degree of aggregation (Fig. 4D). These bands could represent the tetramers and hexamers that were suitable for detection by size exclusion chromatography and MS. In the presence of both Ca and Zn (Fig. 4E) only a single band was visible. Both TPEN (zinc-chelator) and EDTA (divalent ion-chelator), but not BCS (copper I-chelator) inhibit hexamer-formation (Fig. 4F). S100A12 was next pre-treated with TPEN to remove all zinc from the preparations. The protein was then dialyzed back into a Zn-free and TPEN-free buffer, and re-substituted with Zn, Cu, or Ca before loading the gel. There were no tetramers/hexamers in the absence of zinc, which is also not influenced by addition of copper. When re-substituting Zn, there is a tetramer/hexamer formation, which is also induced to a lesser extent by adding calcium (Fig. 4G).

### Size exclusion chromatography

The influence of zinc and calcium on the changes of the oligomerization state of S100A12 was analysed by SEC (size exclusion chromatography). In the absence of the ions the protein eluted as a dimer, with some trace of a tetramer. In the presence of EDTA it was a strict dimer. When the buffer contained 10 μM zinc the protein size corresponded to a tetramer. With 1 mM calcium it was mostly a dimer, but with a "shoulder" of a tetrameric fraction, with higher calcium concentration this "shoulder" reshaped forming a low peak closer to a hexamer, and finally, in the presence of 10 μM zinc only 2 mM calcium was enough for a complete transition to a hexameric form (Fig. 4H).

### ESI-MS analysis of S100A12 and its complexes with the group IIa and IIb metals

We first determined the capillary potential in the ion source required to observe metallo-S100A12 complexes in the gas phase, since excessively high capillary potential causes dissociation of non-covalent complexes. The potential was varied between 33 V and 138 V. At a capillary potential of 138 V no dimer peaks were observed; above 63 V both monomer and dimer peaks were evident (+4, +5 and +6 charge states) and below 48 V no monomer peaks were observed. We chose to use a capillary potential of 48 V or less for subsequent experiments [41].

**Figure 5:**
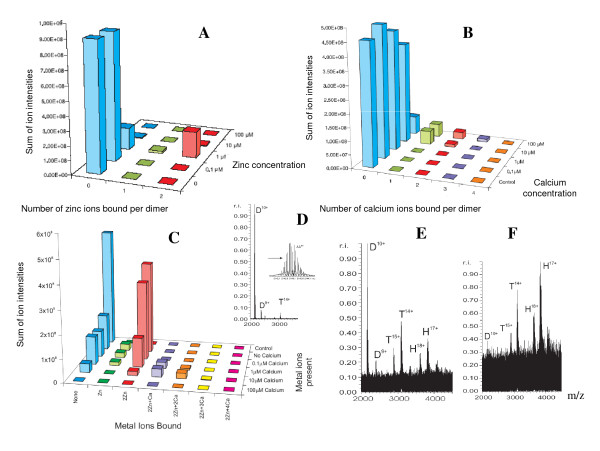
**Mass-spectrometry of S100A12/metal complexes**. Bar charts showing the number of metal ions observed to be bound to the S100A12 dimers when S100A12 was dialyzed against a range of metal ion concentrations and analyzed by ESI FTICR mass spectrometry. (**A**) A range of Zn^2+ ^concentrations; control was a solution with no metal ions; (**B**) a range of Ca^2+ ^concentrations; (**C**) a range of Ca^2+ ^concentrations in the presence of 1 μM Zn^2+^. All charts show the sum of the intensities for each metal ion number, summed over all charge states present. (**D**) ESI FTICR mass spectra of 200 μM S100A12 with no metal ions added; (**E**) 100 μM ZnCl_2 _and 200 μM CaCl_2 _added; (**F**) 200 μM ZnCl_2 _and 400 μM CaCl_2_. D = S100A12 dimers, T = S100A12 tetramers, H = S100A12 hexamers.

Zn^2+^-S100A12 complexes were formed by dialyzing a solution of S100A12 against a range of Zn concentrations. At Zn concentrations of 1 μM or greater, we observed the formation of a fully saturated complex containing two Zn ions per dimer. Dimers containing one Zn ion were also evident, but the intensity of spectra for dimers containing two Zn were much more intense. Dimers containing no Zn ions were also present and the intensity of their spectra were greater than that of dimers containing a single Zn ion (Fig. 5A).

The stability of Zn^2+^-S100A12 dimers was assessed by increasing the capillary potential to 123 V. At this potential, dimers containing two zincs ions persisted, whereas for apo-S100A12, only monomers were evident. Thus, metal-bound complexes are more stable than apo-complexes in the gas phase that has also been previously observed by Alfonso et al [42].

We also examined the binding of Cu^2+^, Ca^2+^, Fe^2+^, Ni^2+ ^and Co^2+ ^ions to S100A12 dimers. Fully saturated complexes were formed at Cu concentrations above 10 μM (not shown) and at calcium concentrations above 100 μM (Fig. 5B). However, adding Fe^2+^, Ni^2+ ^and Co^2+ ^did not result in any new species in the mass spectra (not shown), suggesting that Cu^2+^, Zn^2+ ^and Ca^2+ ^binding to S100A12 dimers is specific.

Next we assessed calcium binding to S100A12 dimers in the presence of 1 μM Zn; at this concentration each S100A12 dimer contained predominantly two Zn ions. At 1 μM calcium, one or two calcium ions were bound to S100A12 dimers, whereas no calcium was bound to apo-S100A12. At a Ca concentration of 10 μM, up to three calcium ions were bound to each S100A12 dimer, and the average number of calcium ions bound was 1.1 per dimer *versus *0.16 per dimer in the absence of Zn ions. When the Ca concentration was increased to 100 μM, no signal was observed, suggesting that larger oligomeric species were formed. The intensity of each species present at different calcium concentrations is shown in Fig. 5C. These data show that bound Zn increases the affinity of the S100A12 dimers for Ca.

To detect oligomeric species in the presence of Ca and Zn ions, we increased the concentration of S100A12 to 200 μM. Adding 100 μM Zn and 200 μM Ca produced S100A12 dimers, tetramers and hexamers (Fig. 5E). Increasing the concentration of Zn and Ca ions to 200 μM and 400 μM (Fig. 5F), respectively, shifted the equilibria towards the higher order oligomers, with hexamers as the dominant species and dimers almost absent from the spectrum. Hexamers were observed in the +16, +17 and +18 charge states, while tetramers were observed in the +13, +14 and +15 charge states. Spectra with no metal ions contained dimers and some tetramers, but no hexamers (Fig. 5D).

The total ion signal intensity decreases with increasing concentrations of metal ions. This decrease correlates with the binding affinity of the ions for S100A12 (data not shown). For example, to decrease the signal intensity by 50%, Zn, which binds to S100A12 with the highest affinity, required the lowest concentration (1 μM) followed by Cu (10 μM) and Ca (100 μM). The most likely explanation for this decrease is protein aggregation. Results using DLS and size exclusion chromatography confirm the presence of highly oligomerised species. Finally, the presence of a substantial population of tetrameric intermediates suggests that formation of S100A12 hexamers may not be cooperative as is the case for the formation of insulin hexamers [43].

### Tyrosine fluorescence titration of S100A12 with bivalent metal cations

**Figure 6:**
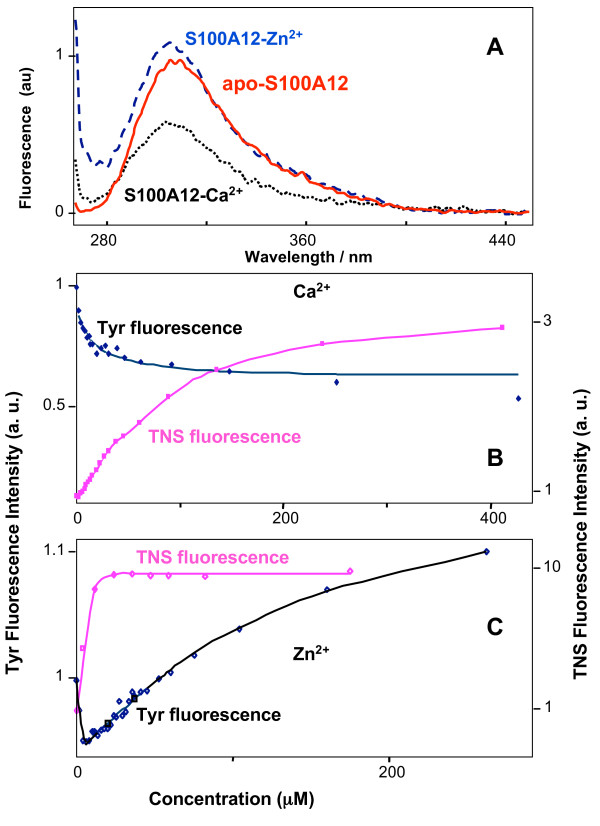
**Tyrosine and TNS fluorescence of S100A12/metal complexes**. (**A**) Intrinsic tyrosine fluorescence spectra of apo-S100A12 (2.5 μM), and S100A12-Ca2+ and S100A12-Zn2+ complexes in 10 mM pH 7.4 Hepes buffer, at total metal ion concentration of 275 μM and 244 μM, respectively. (**B **and **C**) Fluorescence titration of apo-S100A12 with Ca^2+ ^and Zn^2+^.

We used intrinsic protein fluorescence as a sensitive assessment of ion binding and changes in protein conformation. Two tyrosine residues, Y17 and Y86 contribute to the intrinsic fluorescence of S100A12. We monitored the changes in tyrosine fluorescence as a result of titration with Ca^2+^, Zn^2+ ^and a combination of Ca^2+ ^and Zn^2+^. The addition of 50 μM EDTA into the solution of S100A12 did not result in any noticeable change in the protein fluorescence (data not shown) that confirmed the apo-state of the prepared and purified protein. Titration of S100A12 with Ca at pH 7.4 (10-mM HEPES buffer) resulted in a gradual decrease in the tyrosine fluorescence intensity (Fig. 6A, B) with a minor peak shift of 1–2 nm (Fig. 6A). The change in the intrinsic tyrosine fluorescence intensity with Ca^2+ ^was well fit to one-site binding model (eq. 1). Since S100A12 has two Ca^2+ ^sites, we assume that one site does not affect the tyrosine fluorescence. This is in agreement with similar observations we have made earlier [unpublished data] for S100A4 protein and its mutants lacking N-terminal or C-terminal tyrosines. The best fit gave an estimated value of *K *= (5.9 ± 1.7)*10^4 ^M^-1 ^for the equilibrium constant of the Ca^2+ ^binding reaction. This value was higher than the previously reported one, (1.9 ± 0.4)*10^4 ^M^-1 ^for pig granulocytes S100A12 at pH 7.4 in Tris-HCl buffer [31]. For comparison, we repeated the fluorescence titration experiment in 25-mM Tris-HCl (pH 7.4) buffer and obtained the value of (1.2 ± 0.7)*10^4 ^M^-1^, which is consistent with the previously reported result.

In contrast to calcium, S100A12 titration with zinc resulted in non-monotonic changes in tyrosine fluorescence that might indicate the occurrence of at least two different binding sites for this ion. However, (i) the predominant dimeric appearance of apo-S100A12 and (ii) strong protein aggregation in the presence of a small amount of Zn complicates the interpretation of the fluorescence titration data (see Discussion). As shown in Fig. 6C, the peak fluorescence intensity decreases sharply by ~5% at low Zn^2+ ^concentrations (from 2 μM to 6 μM) and increases at higher concentrations reaching a level exceeding the initial intensity by ~10% at sub-millimolar Zn concentrations (intensity changed from 309→295→337 a.u.). It is interesting, that previously, a steady increase in fluorescence intensity for Zn titration curves has been reported for S100A12 obtained from pig granulocytes, and a single high affinity Zn^2+ ^binding site per a monomer has been proposed. A non-monotonic fluorescence change on zinc titration has been reported also for S100A8/A9 proteins [44]. Obviously Zn^2+ ^induced different conformational changes of S100A12 compared to Ca^2+^. Zn^2+ ^titration data fitted well to two-binding site model (eq. 3) with the stability constants of (4.5 ± 0.8)*10^5 ^M^-1 ^and (7.5 ± 1.7)*10^3 ^M^-1^.

**Figure 7:**
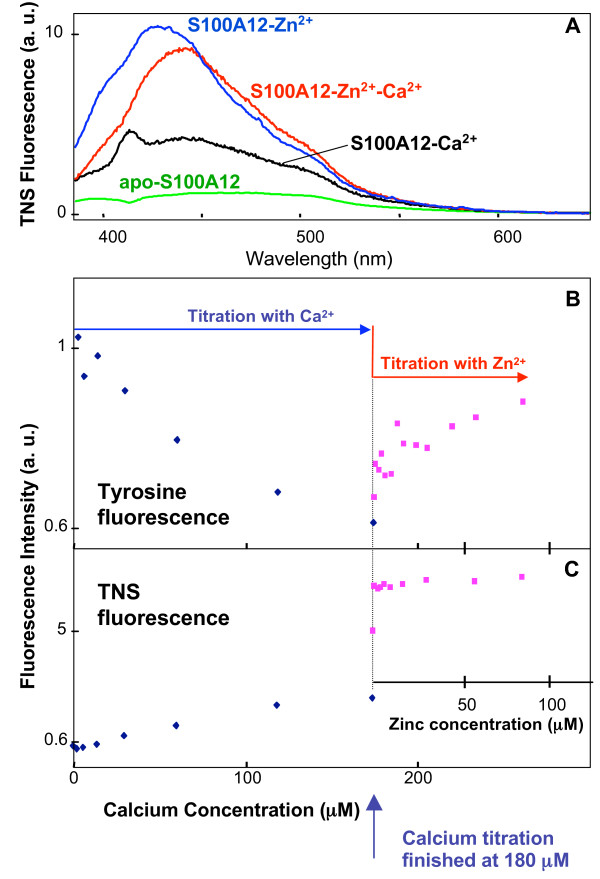
**Tyrosine and TNS fluorescence of S100A12/metal complexes**. (**A**) TNS Fluorescence spectra of S100A12-Ca^2+^, S100A12-Zn^2+ ^and S100A12-Zn^2+^-Ca^2+ ^complexes in 10 mM pH 7.4 Hepes buffer, at total metal ion concentration of 180 μM (Ca^2+^) and 200 μM (Zn^2+^). (**B **and **C**) Fluorescence titration of apo-S100A12 first with Ca^2+ ^up to the total concentration of 180 μM and then with Zn^2+^. S100A12 concentration was 2.5 μM, and TNS concentration was 20 μM.

**Figure 8:**
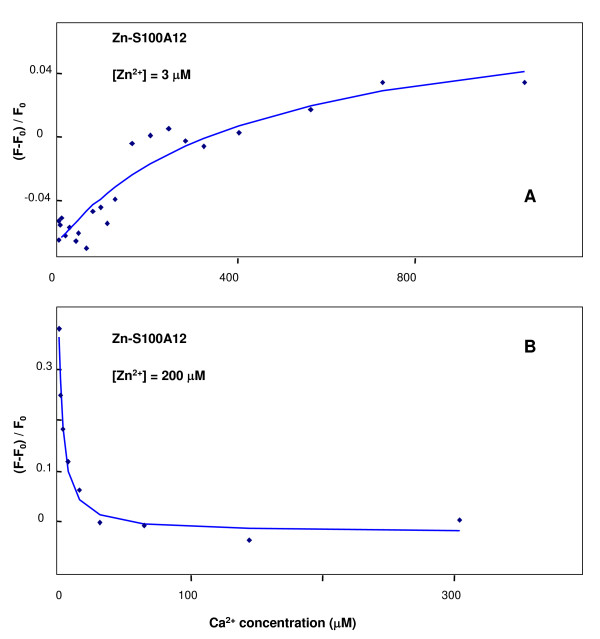
**Intrinsic tyrosine fluorescence titration**. of S100A12-Zn^2+ ^with Ca^2+ ^in 10 mM pH 7.4 Hepes buffer. S100A12 concentration was 2.5 μM.

Interestingly, the initial decrease in the fluorescence intensity at low Zn^2+ ^concentration (2 μM-6 μM) in the absence of Ca^2+ ^(Fig. 6C) disappeared in the presence of 180 μM Ca^2+^(Fig. 7B). Upon addition of Zn^2+ ^to the Ca-loaded S100A12, a gradual increase was observed (Fig. 7B), which is consistent with the previous result reported for Zn^2+ ^titration to pig granulocytes apo-S100A12 [31]. The reverse titration of Ca^2+ ^to Zn^2+^-loaded protein at low (3 μM, Fig. 8A) and high (200 μM Zn^2^, Fig. 8B) showed diverse effects. Sharp decrease in the fluorescence intensity (Fig. 8B) was observed when protein was fully saturated with zinc, which is consistent with the previous result that Zn^2+ ^increases Ca^2+ ^affinity to pig granulocytes S100A12 protein. Calcium titration curve for the protein at low zinc concentration was completely different, with a very gradual increase of the intensity (Fig. 8A); this could be interpreted as weak calcium binding to the aggregated protein. It is noteworthy here that we are not reporting on the quantitative aspects of Ca^2+ ^and Zn^2+ ^binding (exact number of binding cations per protein molecule) but presenting qualitative fluorescence data, which show that the presence of zinc ion can dramatically change the affinity and fluorescence response to calcium ion.

### TNS fluorescence titration of S100A12 with metal ions

A large increase in TNS fluorescence intensity was observed on titration of apo-S100A12 with metal ions, however, Zn^2+ ^induced a much larger change than calcium. In addition, the TNS fluorescence peak showed a blue shift in the presence of Zn relative to the fluorescent peaks obtained in the presence of calcium (Fig. 7A). The latter may indicate the accessibility of a larger hydrophobic surface to TNS upon Zn^2+ ^binding.

In contrast to the intrinsic tyrosine fluorescence, TNS indicated a one-step reaction only on S100A12 titration with Zn^2+^. TNS florescence intensity increased sharply when Zn^2+ ^concentration increased from ~1 μM to ~6 μM and did not change upon further Zn^2+ ^addition (Fig. 6C). In other words, TNS was sensitive to the S100A12 structural rearrangements caused by the binding of the first cation, but showed no changes due to the binding of a second Zn ion. However the binding of the second Zn^2+ ^ion resulted in a substantial increase in the intrinsic tyrosine fluorescence intensity. The addition of a small amount of Zn^2+ ^to Ca^2+^-loaded S100A12 did result in protein structural changes and the appearance of an accessible hydrophobic surface since the TNS fluorescence showed a sharp change. Furthermore, the observed blue shift of TNS fluorescence peak upon Zn^2+ ^binding to apo S100A12 protein disappeared in the case of Zn^2+^-Ca^2+^-S100A12 complex (Fig. 7A). A TNS fluorescence titration of Zn^2+^-S100A12 complex with Ca^2+ ^confirmed this observation by showing the obvious red shift upon addition of Ca^2+ ^with no intensity change (data not shown). These results indicated that the TNS binding sites formed as a result of complex formation between S100A12 and Zn^2+ ^alone was different from those between S100A12, and Zn^2+ ^and Ca^2+ ^together.

### Mapping of the Zn^2+ ^binding surface by NMR spectroscopy

Ca^2+^-S100A12 was extensively studied by NMR spectroscopy [20]. We decided to further utilize this technique to obtain atomic resolution information on the structural elements of Ca^2+^-S100A12 involved in binding Zn^2+^.

NMR chemical shift perturbations technique is exquisitely sensitive to the changes in the protein structure upon complex formation. Depending on the nature of the molecular interactions, protein complex formation may result in either changes in the position of the NMR peaks or their broadening. Both changes can be mapped onto the three-dimensional structure of the protein delineating the interaction surface.

**Figure 9:**
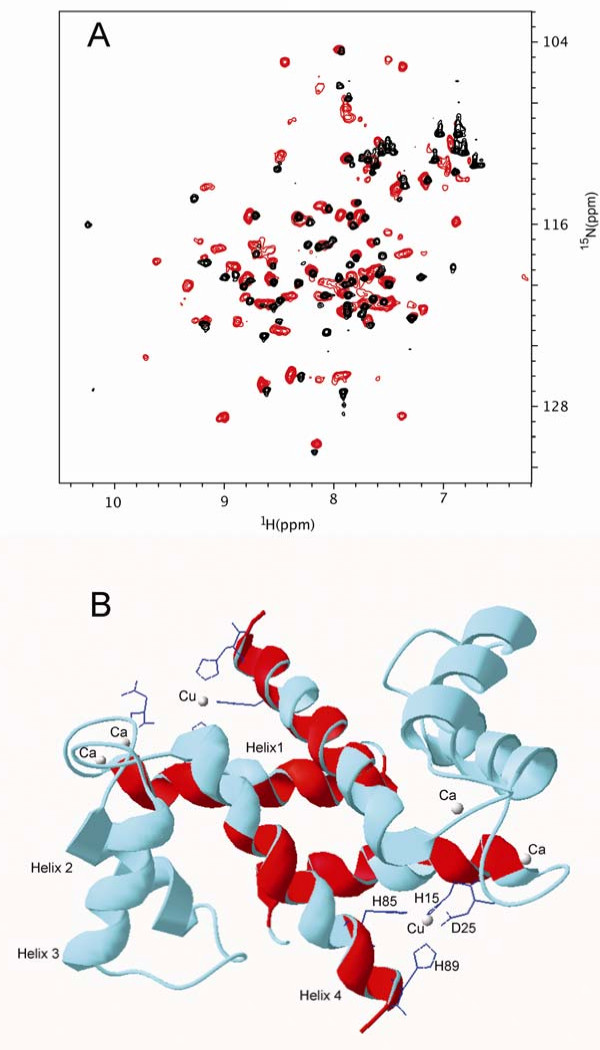
**Mapping of the interaction surface involved in the Zn^2+^Ca^2+^-S100A12 complex formation using NMR chemical shift perturbations**. (**A**) Overlay of the ^1^H{^15^N}-HSQC spectra of [*U*-, ^15^N] Ca^2+^-S100A12 (black) and [*U*-, ^15^N] Zn^2+^, Ca^2+^-S100A12 (red) shows that 54 out of 80 amide peaks do not significantly change their position upon addition of Zn^2+ ^to Ca^2+^-s100a12. Significant spectral broadening of the Zn^2+^, Ca^2+^-S100A12 peaks is due to the increased molecular weight of the oligomer. (**B**) Amino acids of the Zn^2+^, Ca^2+^-S100A12, whose amide peaks are either completely broadened or underwent large (> 0.1 ppm) chemical shift change, are mapped onto the backbone of crystal structure of the Cu^2+^, Ca^2+^-S100A12 dimer (PDB code 1ODB. They are shown in red and cluster around Cu^2+ ^binding motif suggesting that this motif is also involved in  Zn^2+^-binding. Amino acids that are not involved in Zn^2+^-binding are shown in cyan.

Titration of [U-, ^15^N] Ca^2+^-S100A12 with Zn^2+ ^ion resulted in the dramatic uniform increase of the line broadening of the ^1^H{^15^N}-HSQC spectrum. (Fig. 9A). This suggests that the protein complex with Zn^2+ ^forms large aggregates, consistent with our previous observation. After the metal:protein ratio (w/w) reached 1.3:1, there were no more changes in the NMR spectrum. This ratio is consistent with one Zn^2+ ^ion binding to Ca^2+^-S100A12 monomer observed in our ESI-MS study.

In spite of the obvious increase in the size of Zn^2+^Ca^2+^-S100A12 compared to Ca^2+^-S100A12, the majority of its amide protons and nitrogens did not change significantly their chemical shifts. 53 out of 82 amide hydrogen and nitrogen peaks observed in ^1^H{^15^N}-HSQC spectrum of [U-, 15N] Ca^2+^-S100A12 changed their chemical shifts less than 0.05 ppm. This observation suggested that complexation with Zn^2+ ^did not result in dramatic change of the overall structure of Ca^2+^-S100A12 dimer. Structural elements that did not undergo significant changes due to binding to Zn^2+ ^include loop I, helix II, loop II, helix III and linker loop. Since these elements are involved in binding Ca^2+^, we assumed that Ca binding sites were largely intact in Zn^2+^, Ca^2+^-S100A12. Amide peaks that underwent either large chemical shift changes (more than 0.1 ppm) or disappeared due to broadening are located in two structural elements: helix I and helix IV. These elements formed a contiguous surface when we mapped them on the ribbon diagram of Cu^2+^, Ca^2+^-S100A12 dimer (Fig. 9B). The same structural elements are involved in the binding of Cu^2+ ^ion in the Cu^2+^, Ca^2+^-S100A12 crystal structure. Thus, NMR data provided strong evidence that Zn ion bound to the Cu^2+ ^binding site. We may also expect that structural changes associated with Cu^2+ ^binding, extension of the helix 4 and restructuring of the target binding surface, would be similar for binding Zn^2+^.

## Discussion

Our *in vitro *experiments highlighted how the oligomerization of S100A12 can be modulated by the binding of Ca and Zn ions. The results revealed that Zn binding induces a significant change in protein quaternary structure. The dramatic effect of Zn ions on the S100A12 conformational changes provides a rationale for the diverse target binding capability of S100A12 protein.

It was found that S100B bound its target peptide TRTK-12 both in a Ca loaded mode and in a Zn^2+^-Ca^2+^-mode, but not in the complex with Zn [45]. It was proposed that the protein did not adopt the right conformation to recognize this target in the presence of Zn^2+ ^alone. It seemed likely that Ca binding specifically arranged the target-binding region into a shape which enhanced target binding affinity and that these local conformational changes were critical for target protein binding.

The information obtained in our previous studies indicated the importance of the relative position of helices III and IV for target protein binding [46]. NMR chemical shift mapping showed that zinc binding led to the restructuring of helices I and IV, and induced the exposure of a number of hydrophobic residues. In fact, the location of the chemical shift changes due to Zn binding matched that involved in Cu binding.

**Figure 10:**
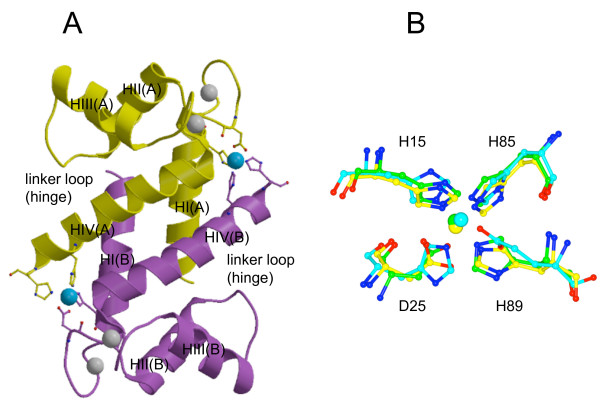
**Comparison of zinc and copper binding sites in S100 proteins**. (**A**) Human S100A12 dimer in the copper-bound state (PDB code 1ODB). Ribbons of the two subunits are in yellow and purple. Calcium ions are shown in grey and copper ion in cyan. Copper-binding residues are shown in ball and stick. (**B**) Superposition of the copper (and potentially zinc) binding site in S100A12 on zinc-binding sites of S100A7 (PDB code 2PSR) and S100B (PDB code 3CR2). Copper-binding residues of S100A12 and copper ion are in yellow, corresponding residues and zinc are in green for S100A7 and in cyan for S100B. The structures were superimposed using the program LSQKAB [59]. Part A of the figure was generated using MOLSCRIPT [60] and rendered with Raster3D [61], part B with CCP4mg [62]

We predicted that a pair of Zn binding sites situated within a dimer would be located at the same position where copper binds in the S100A12-copper structure (PDB code 1ODB; Fig. 10A, B). These sites had a high sequence-structure similarity with the zinc binding sites of several other S100 proteins. A single Zn^2+^- binding site per monomer was first identified for porcine S100A12 by fluorimetric titration, and a C-terminal HisxxxHis Zn-binding motif was proposed based on sequence analysis and secondary structure prediction [31]. The crystal structure of S100A7 revealed the location of the Zn-binding site, which was composed of an N-terminal histidine and an aspartic acid from one subunit and two C-terminal histidines from a second subunit (Fig. 10B). Sequence comparison suggested that S100A8, S100A9, S100A12 and S100B could have similar Zn-binding sites [24]. Involvement of the C-terminal HisxxxHis motif of S100A8, S100A9 and S100A12 in zinc binding had previously been predicted on the basis of sequence alignment [5]. In addition, based on sequence/structure comparisons, it is possible that S100A6 bound zinc in a similar way, although it lacked one of the C-terminal zinc-binding residues.

The same binding site was identified in the NMR and X-ray structures of zinc-loaded S100B [25,47] (PDB codes 1XYD, 3CR2; Fig. 10B). Binding of Zn^2+ ^and Cu^2+ ^resulted in some similar conformational changes upon formation of Zn^2+^-S100B and Cu^2+^-S100A12 complexes. Significant changes at the putative target-binding site upon copper binding were observed for S100A12 protein, including the extension of the C-terminal helix and closer contacts between dimers [46]. These changes were also observed for S100B upon Zn^2+ ^binding. However, no changes in the interhelical angle similar to those observed in S100B-Ca^2+^-Zn^2+ ^system were found upon addition of Cu^2+ ^to the S100A12-Ca^2+ ^complex. Thus, the binding of copper and zinc may result in different changes in the target binding site.

TNS fluorescence data have demonstrated a significant difference between three ion loading modes of S100A12. In particular, a blue shift in the TNS fluorescence peak was found for Zn^2+^-S100A12 complex which was not evident for either Ca^2+ ^nor Zn^2+^-Ca^2+ ^bound proteins. Hydrophobic amino acid residues responsible for this blue shift might contribute to different conformations of Zn^2+^-S100A12 and Zn^2+^-Ca^2+^-S100A12. We tentatively attribute the blue shift in TNS fluorescence to a stronger hydrophobic environment experienced by the TNS molecules bound to Zn^2+^-S100A12 complexes. In the case of Zn^2+ ^bound S100A12, analysis of the amino acid sequences of Helix III and Helix IV, the linker loop, the C-terminus and the N-terminus of the other subunit, all of which contribute to the target binding site, pointed to Lys residues with long hydrocarbon chains as the possible candidates for contributing to the blue shift of the TNS fluorescence peak. A huge observed increase in TNS fluorescence upon Lys-TNS binding in water (data not shown) caused us to conclude that the orientation between Helix III and IV, which buries certain Lys residues, may be critical for target protein binding. These could be the Lys residues from the linker loop (or "hinge" region) – region between helices III and IV, the importance of which for S100A12 function has recently been confirmed [13].

Tyrosine fluorescence titration experiments suggested the presence of at least two Zn-binding sites in S100A12 with significantly different affinities. However, a large increase in TNS fluorescence was evident only at the first stage of titration with Zn. Our previous studies indicated that TNS was more sensitive to aggregation due to hydrophobic interactions than just the appearance of accessible hydrophobic surfaces [20]. Therefore, TNS fluorescence titration might indicate a strong aggregation of S100A12 at low Zn^2+ ^concentrations. We hypothesize that even one Zn^2+ ^per dimer is sufficient to induce the aggregation. Binding a second Zn^2+ ^per dimer occurs under aggregated conditions and might exhibit a different affinity despite the binding site (considering the S100A12 monomer) being the same. This hypothesis agrees well with both NMR (see Fig. 9B and figure legend) and ESI-MS data (Fig. 5).

**Figure 11:**
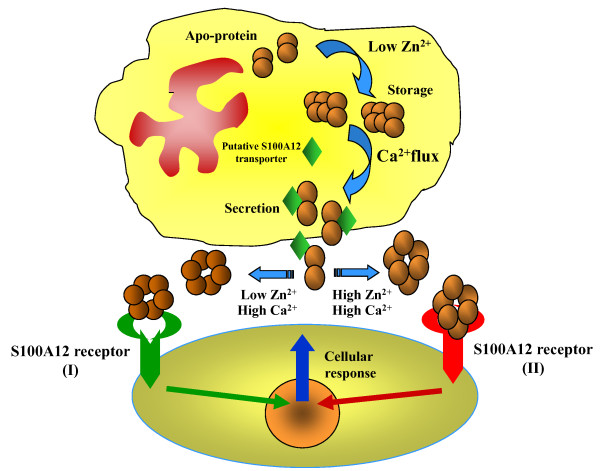
**Graphical representation of hypothetical mechanism on the effect of calcium and zinc on the S100A12 assembly, translocation and interaction with extracellular targets**.

Our surface plasmon resonance studies revealed that indeed the interaction of S100A12 with its cell surface receptor RAGE was facilitated with increasing Ca and Zn concentrations, likely due to the fact that under these conditions more oligomers formed which could bind to RAGE. Available data *in vitro *and *in vivo *open the possibility that the cellular pool of Zn may be sufficient to bind to S100 proteins. Zn ions are generally tightly bound by the proteins [48,49] and it has been proposed that Zn is normally transferred directly from a donor to an acceptor molecule. However recent studies have demonstrated the importance of weakly bound Zn [50]. The physiological significance of potential intracellular S100A12 aggregation in the presence of very low concentrations of zinc ions has a rational explanation. In resting neutrophils the level of Ca is very low and is not enough to saturate any of the S100 proteins with Ca. The binding of zinc to S100A12 may lead to its localization in large oligomeric form (larger than hexamers), thus preventing the free distribution of S100A12 within the cytoplasm and leading to its accumulation in specific locations. In addition, the presence of Zn may increase the sensitivity of S100A12 to Ca up to 1500 fold. This phenomenon has been confirmed for both human and pig S100A12. Ca import into cells activates S100A12 to bind to its putative transporter and/or to other intracellular targets and to translocate toward the cellular membrane (Fig. 11). The calcium activated translocation has been demonstrated by Guignard et al[4]. A very interesting hypothesis [32] based on the earlier work [51,52] has been suggested; that at low oxidative stress several Zn-binding proteins may release free Zn and that this Zn is able to regulate the activity of S100 proteins. Under oxidative conditions in neutrophils, it is possible that Zn ions released from the cysteine-containing Zn binding proteins could be accepted by other proteins with histidine Zn binding motifs. Due to the high level of expression in neutrophils S100A12 may be a good acceptor of escaped zinc. We proposed that S100A12 is also regulated in the same manner by binding various amounts of zinc and changing its oligomeric status. On the other hand, the binding of S100 proteins to Zn attenuates a potential toxicity by sequestering Zn ions. Outside the cell where Zn concentration depends on the local microenvironment and the Zn level can rapidly rise and fall, all types of S100A12 oligomers may exist in equilibrium depending on the local Zn concentration. This may explain how S100 proteins are managed to specifically interact with different extracellular receptors supporting and regulating signal transduction pathways (Fig. 11).

Oligomeric S100A12 species have been discovered in blood serum [53] and in human tissues [17]. The latter study investigated the presence and deposition of S100A12 and other family members in familial (PS-1) and sporadic AD cases. In all cases 70-kDa S100A12 species have been found. The higher affinity of oligomeric S100A12 to RAGE demonstrated in our recent and current studies could explain the elevated level of S100A12 high-molecular weight species in AD brain samples. The changing extracellular environment is a mechanism that may effectively modulate the structural properties of S100A12 and its ability to bind RAGE.

The existence of zinc binding sites in S100 proteins that are able to transmit signals through RAGE and other putative extracellular receptors raises a very important question about the role of S100 proteins in the cross-talk between zinc and calcium. In this model the role of S100 signalling is likely to be similar to the role of Zn sensing receptors. S100 signals could be accepted by extracellular receptors coupled with G-proteins, with further activation of PLC and a rise in Ca concentration. It was demonstrated that the cellular response to S100A12 was abolished by inhibitors of phospholipase C (PLC), protein kinase C (PKC), Ca^2+ ^flux and Ca^2+^/calmodulin dependent kinase II [54].

This hypothesis provides a simple integration mechanism that is able to transmit a signal from the cellular microenvironment when local changes of Zn concentration and pH are generated by physiological and pathophysiological events.

## Methods

### Protein Expression and Purification

#### S100A12

Recombinant full-length human S100A12 was expressed in *E. coli *from the modified pQE60 vector containing S100A12 gene. Although the pQE60 vector contains a 6×His sequence a stop codon was deliberately inserted so that the protein was expressed without a histidine tag. Protein expression was induced with 1 mM IPTG and the bacterial pellet was lysed by sonication. After sonication the insoluble material was removed by centrifugation. The supernatant was adjusted up to 10 mM CaCl_2 _and after 15 min incubation at room temperature was clarified by centrifugation. Clear supernatant was applied onto Phenyl-Sepharose column equilibrated with 50 mM Tris-HCl (pH 7.5), 150 mM NaCl, 5 mM CaCl_2_, the protein was eluted with 50 mM Tris-HCl (pH 7.5), 150 mM NaCl, 5 mM EDTA. S100A12 containing fractions were concentrated by ultrafiltration in an Amicon centrifugation filter unit (Millipore) buffer-exchanged into 50 mM Tris-HCl pH 7.9 buffer, and applied to a 1 ml ResQ column (Pharmacia), equilibrated with 50 mM Tris-HCl pH 7.9 buffer. The protein was eluted with a 0–1 M NaCl gradient using an AKTA Purifier chromatography system (Amersham). Protein concentration was determined by BCA protein assay (BioRad). After purification a single 10 kDa band was observed on the SDS-PAGE.

#### Extracellular domain of RAGE

Recombinant soluble extracellular fragment of human RAGE (aa 24–336) was purified according to recently described protocol [20].

The molecular masses of the purified proteins were verified by mass spectrometry (MALDI) giving peaks of 37213 Da for RAGE with His-tag, 35190 Da for RAGE with cleaved His-tag and 10642 Da for S100A12. These values are in close agreement with the calculated molecular masses.

### Near-Field Scanning Optical Microscopy (NSOM)

The human gastric cancer cell line MKN74 was obtained from the Health Science Research Resources Bank (Japan Health Sciences Foundation, Osaka). MKN74 is a cell line established from moderately differentiated adenocarcinoma.

MKN74 cells were cultured on 18-mm glass coverslips for 16 h, then washed with a fresh medium without serum and incubated in the presence of 10 μg/ml S100A12 at 10°C in F12 and RPMI 1640 cell culture medium without serum. After incubation cells were washed three times with PBS (pH 7.4), fixed with 3% paraformaldehyde, 2 mM MgCl_2 _and 4% sucrose at room temperature for 20 min, incubated in 0.15 M glycine in PBS and washed again with PBS. Then one set of coverslips was incubated in PBS and served as the no-primary antibody control. Additional control was used in the absence of exogenous S100A12. Both controls did not reveal any clusters on the cellular surface. The experimental set was incubated in the presence of 5 μg/ml rabbit affi-pure anti S100A12 antibody at room temperature 45 min. The antibody solution was aspirated and the cells were washed three times for 3 min with PBS. All coverslips were then incubated with secondary chicken anti-rabbit Alexa488 labelled antibody (Invitrogen/Molecular Probes) at room temperature 45'- 1 h. For NSOM imaging, after the three washes with PBS, the cells on 18-mm coverslips were rinsed three times with distilled autoclaved water and allowed to air-dry for a minimum duration of 12 h before imaging.

Bent NSOM probes were prepared from high GeO_2_-doped fibers with a core diameter of 3 μm using a two-step chemical etching method followed by aluminum deposition and focused ion beam milling to produce a flat circular aperture [55,56]. Probes with aperture diameter of ~100 nm were used in the present work (estimated from SEM images). The estimated spring constant for these probes is ~100 N/m [55].

NSOM experiments were carried out on a combined AFM/NSOM microscope based on a Digital Instruments Bioscope mounted on an inverted fluorescence microscope (Zeiss Axiovert 100). A separate x-y piezo scanner (Polytec PI, Auburn, MA, 50 nm lateral scan range) was used for sample scanning. A continuous wave mixed gas ion laser (Coherent, Innova 70 Spectrum) was used for excitation purposes (488 or 568.5 nm, 20 mW, linear polarization). NSOM signal was collected with 63 × (0.75 NA) objective, with appropriate band pass (Omega Optics) and notch filters (Kaiser Optical Systems, Ann Arbor, MI) to remove residual excitation and red alignment laser light and detected using an avalanche photodiode detector (Perkin Elmer Optoelectronics, SPCM-AQR-15, Vaudreuil, Canada). Images were recorded in tapping mode at a scan rate of 0.25 Hz and a resolution of 512 × 512 pixels. Images were processed using standard Nanoscope software as well as Image J software (NIH). Cluster size analysis was performed using original non-processed NSOM images with custom-made software. The software allows the determination of the number of clusters, their location in the image, as well as their height (intensity) and halfwidth.

### Immunofluorescence Microscopy

For S100A12 immunofluorescence microscopy, MKN74 cells were cultured in a 8-well LAB-TEK^® ^Permanox chamber slide (Nalge Nunc International, Naperville, IL) for 16 h, then washed with a fresh medium without serum and incubated in the presence of 10 μg/ml S100A12 at 10°C in F12, F12K or RPMI 1640 cell culture medium without serum. (F12 medium contains Zn2+ [3.5 μM] and Ca2+ [0.3 mM] and Cu2+ [10 nM]; F12K medium contains Zn2+ [0.6 μM] and Ca2+ [0.9 mM] and Cu2+ [9 nM]; RPMI 1640 medium contains Ca^2+ ^[0.55 mM]. No zinc and copper).

After incubation cells were washed with PBS (pH 7.4), fixed with 3% paraformaldehyde, 2 mM MgCl_2 _and 4% sucrose at room temperature for 20 min, incubated in 0.15 M glycine in PBS and washed again with PBS. The cells were then blocked with 7% serum in PBS at room temperature and incubated with affinity purified rabbit anti-S100A12 (kindly provided by A Larsen) in PBS containing 1% serum for 1 h, followed Alexa-594 labeled chicken anti-rabbit IgG (Molecular Probes, Invitrogen) diluted 1:1000. The specimens were washed and mounted using Dako fluorescent mounting medium. Double-labeling immunofluorescence was visualized using Carl Zeiss Laser Scanning Confocal System (LSM 510, equipped with a C-Apochromat 63 ×/1.2 W corr objective).

### Fluorescence

LS-55 luminescence spectrometer from Perkin Elmer was used for fluorescent studies. The measurements were made in a 1 × 1 cm·cm quartz cell at 25°C. The resulting titration curves were analyzed using SPSS (Lead Technologies Inc.) and Mathcad 2001 Professional (Mathsoft Inc.) software.

Tyrosine fluorescence was measured using 255-nm excitation keeping the excitation and emission slits at 5 and 10 nm, respectively. For the metal titration experiments, S100A12 solutions were prepared at 2.5 μM concentration in pH 7.4 (for both Ca^2+ ^and Zn^2+^) and 10 mM HEPES buffer. Ca^2+ ^(2 mM), Zn^2+ ^(2 mM) stock solutions were added to S100A12 samples to get final metal concentrations of 400 μM, and 50 μM, respectively. Zn^2+ ^titration of Ca^2+^-S100A12 complex solution was performed at pH 7.4, 10 mM HEPES buffer. 365-nm excitation and 5/8-nm excitation/emission slits were used for all the TNS fluorescence measurements. All solutions for titration experiments were prepared in the same way as those for tyrosine fluorescence measurements except that 20 μM of TNS was added to the 2.5-μM S100A12 solutions.

### Data analysis

The fluorescence intensity (*F*) was corrected for sample dilution. The apparent association constants *K *of Ca^2+ ^with S100A12 were calculated by fitting the relative fluorescence intensity *F *to the function (Eq. 1)

(1)

(2)

derived from a one-site-binding model (Eq. 2) using SPSS program, in which *f*_1 _is the fluorescence at zero ligand concentration, *f*_2 _is the fluorescence of the complex, *N *is the initial protein concentration, *W *is the total metal ion concentration.

The apparent association constants *K *of Zn^2+ ^with S100A12 was calculated by fitting the relative fluorescence intensity *F *to the function (Eq. (3) derived from a two-site-binding model (Eq. (4) using SPSS program.

(3)

(4)

Where *f*_1 _is the fluorescence of the protein at zero ligand concentration, *f*_2 _is the fluorescence of complex PM, *f*_3 _is the fluorescence of the complex PM_2_, *K *is the association constant of the complex PM, *L *is the association constant of the complex PM_2_. *M *is the total metal ion concentration.

### SPR RAGE-S100A12 binding assay

Human RAGE-Fc fusion protein (R&D Systems Inc. Minneapolis, MN) or alternatively recombinant human S100A12 was immobilized by amine coupling at low density on a carboxymethylated dextran-coated CM5 gold sensor surface. Recombinant human S100A12, overexpressed in *E. coli *was analyzed for binding to immobilized RAGE in 10 mM HEPES, pH 7.4, and 0.1 M NaCl, and different concentrations of Zn^2+ ^and Ca^2+ ^using the surface plasmon resonance (SPR) technology (BIAcore 3000 from Biacore, Uppsala, Sweden). Interactions were monitored for 3 min using a flow rate of 30 μl/min. The sensor surface was then washed with the same buffer to start the dissociation for 3 min, and the chip was finally regenerated with a 30 s pulse of 10 mM glycine-HCl, pH 2.1 at 30 μl/min. To calculate affinity and on-/off-rates of the S100A12/RAGE interaction human RAGE/Fc was injected over immobilized S100A12 in a HBS-P buffer containing 1 mM Ca^2+ ^and 20 μM Zn^2+^. The sensorgram obtained was fitted to a 1:1 Langmuir model (BIAevaluation software; Biacore).

### Native Gel Electrophoresis

Native (nondenaturing) polyacrylamide gel electrophoresis (PAGE) was performed at a constant voltage (100 V) with a Hoefer electrophoresis system using a Tris-HCl 7.5% polyacrylamide resolving gel, and Tris-Glycine running buffer, pH 8.8. Samples were prepared in the absence or presence of zinc and calcium. In the presence of zinc and calcium alternatively the zinc-chelator NNNN-tetrakis (2-pyridylmethyl) ethylenediamine (TPEN), the divalent ion chelator ethylenediaminetetra-acetic acid (EDTA), or the copper I-chelator bathocuproine sulfonate (BCS) were used to deplete the respective metal ions from solutions. All chelating agents were purchased from Sigma, Schnelldorf, Germany. Gels were stained with Coomassie R250.

### Size exclusion chromatography (SEC)

Gel-filtration chromatography was carried out using Superdex 200 HP 10/30 column (Amersham Pharmacia Biotech) controlled by an AKTA FPLC system (Amersham Pharmacia Biotech) and UNICORN 5.0 software. The column was equilibrated with 2 column volumes of gel filtration buffer (200 mM NaCl, 50 mM Tris-Hcl pH 7.5) in the presence or absence of different concentrations of zinc and/or calcium. The column was run at 20°C at 0.6 ml/min. Molecular markers used for column calibration were 19.7 KDa (chymotrypsinogen), 45.8 KDa (ovalbumin) and 67 KDa (albumin). The concentration of the injected protein was 1 mg/ml, injection loop size 200 μl. The eluate was monitored by absorbance at 215 and 280 mm and the collected fractions analyzed by SDS-PAGE.

### Mass spectrometry

All mass spectrometric measurements were made on a Bruker (Bruker Daltonics, Billerica, MA, USA) APEX II 9.4 tesla FTICR mass spectrometer, which has previously been described [57]. The ESI source used was from Analytica (Analytica of Branford, Branford, USA). The needle that contained the analyte solution was positioned off-axis; carbon dioxide was used as a nebulising gas. The solution was pumped at a flow rate of 100 μl hour^-1^. Carbon dioxide heated to 250°C was used as a drying gas.

S100A12 protein was prepared for ESI mass spectrometry by desalting over a PD10 column (Amersham Biosciences) using a 10 mM ammonium acetate solution. The protein concentration was calculated using results of the Bradford reagent reaction. 20 μl of the S100A12 solution was placed in a Slide-A-Lyzer MINI dialysis unit and dialysed overnight against a large volume of solution at the desired metal-ion concentration. Before analysis the solution in the MINI-dialysis unit was removed and the protein solution diluted to the desired concentration using metal ion solution from the volume used for the dialysis.

### NMR experiments

NMR experiments were performed on Avance Bruker spectrometer, operating at a ^1^H frequency of 700 MHz and equipped with a cryoprobe. All NMR data were collected at 25°C. Protein samples of S100A12, with concentrations ranging from 0.3–0.5 mM were dissolved in NMR buffer (10 mM Hepes/NaOH (pH 6.5), 100 mM NaCl, 0.02% (w/v) NaN_3_.). To prepare Ca^2+^-S100A12, a solution of 1 M CaCl_2 _was titrated into a 0.5 mM [U-^15^N] S100A12 solution until the molar ratio of metal:protein was 6:1. No changes in the NMR spectrum of the protein were detected at higher molar ratios. To prepare Zn^2+^Ca^2+^-S100A12 a solution of 0.5 M ZnCl_2 _was titrated into 0.5 mM solution of Ca^2+^-S100A12 until the molar ratio of metal:protein was 3:1. No changes in the NMR spectrum of the protein were detected at higher molar ratios. Gradient diffusion experiments to obtain translational diffusion coefficient, D, were performed using pulse sequence described in [58].

This experiment measures the attenuation of the NMR signal due to the increase in the strength of the gradient field and can be used to calculate translational diffusion coefficient, which is for the spherical Brownian particle inversely proportional to Stokes' radius

(5)

*K*_*B*_*T *being the thermal energy, *η *the viscosity of the solvent and *r*_*a *_Stokes' radius. In our experiment the diffusion delay was Δ + 6τ = 1 s; each sine-shaped encoding gradient lasted δ = 1.3 ms. Translational diffusion coefficient was calculated by fitting integrated amide signal intensities using the equation:

(6)

where

(7)

γ is the proton gyromagnetic ratio, *s *the shape of the encoding and decoding gradient pulses, and *G*_max _their peak amplitude.

### Structure comparisons and molecular graphics

The structures were superimposed using the program LSQKAB [59]. Part A of Fig. 10 was generated using MOLSCRIPT [60] and rendered with Raster3D [61], part B with CCP4mg [62].

## Abbreviations

Zn: (zinc); Ca: (calcium); Cu: (copper); AEBSF: 4-(2-aminoethyl)-benzenesulfonyl fluoride; TNS: 6-(p-toluidinyl) naphthalene-2-sulphonic acid; AFM: (atomic force microscopy); DLS: (dynamic light scattering).

## Authors' contributions

OVM participated in the experiment design, carried out biochemical and structural analysis, co-wrote and helped to finalize the manuscript, WB performed MS-ESI- FTICR experiments, contributed to the new analytical tools and data analysis, HW designed and carried out the SPR experiments, analyzed the data and co-wrote the manuscript, WH performed the fluorescent spectroscopy experiments and data analysis, and contributed to drafting of the manuscript, AI designed and performed NSOM experiments, analyzed the data, contributed to new analytical tools and participated in drafting of the manuscript, VN designed and performed the fluorescent microscopy experiments and analyzed the data, JX carried out NMR experiments and data analysis, OP carried out biochemical analysis, IKL designed and performed fluorescent spectroscopy experiments and data analysis, co-wrote the manuscript,^, ^AS designed and supervised NMR experiments, analysed the data and co-wrote the manuscript, PJD carried out data analysis for MS-ESI-FTICR and co-wrote the manuscript, PB and DF designed and performed SPR experiments and data analysis, DF co-wrote the manuscript, IBB conceived of the study, participated in its design and coordination, carried out biochemical and structural analysis, drafted and finalized the manuscript. All authors participated in the discussion and approved the final manuscript.

## Footnotes

**VN **Present address: Institute for Cancer Studies, University of Birmingham, Edgbaston, Birmingham B15 2TT, UK; **WB **Present address: LGC, Queens Road, Teddington, London, TW11 OLY, UK; **PJD **Present address: Institute of Fundamental Sciences, Massey University, Private Bag 11–222, Palmerston North, New Zealand.
